# Interstitial lung changes and persistent COVID‐19 in a patient with follicular lymphoma: A case report

**DOI:** 10.1002/rcr2.1298

**Published:** 2024-02-20

**Authors:** Makiko Yomota, Masaru Tanaka, Takayuki Kobayashi, Masatake Kitano, Saori Ikeda, Yusuke Kanemasa, Noriyo Yanagawa, Yukio Hosomi

**Affiliations:** ^1^ Department of Respiratory Medicine Tokyo Metropolitan Komagome Hospital Tokyo Japan; ^2^ Department of Infectious Diseases Tokyo Metropolitan Komagome Hospital Tokyo Japan; ^3^ Department of Oncology Tokyo Metropolitan Komagome Hospital Tokyo Japan; ^4^ Department of Radiology Tokyo Metropolitan Komagome Hospital Tokyo Japan

**Keywords:** COVID‐19, lymphoma, pneumonitis, tacrolimus

## Abstract

We herein report a case of interstitial lung changes in a patient with prolonged coronavirus disease 2019 (COVID‐19) with follicular lymphoma receiving rituximab and bendamustine who recovered after treatment with a combination therapy consisting of corticosteroids and immunosuppressive agents. There is currently no treatment strategy for prolonged pneumonitis following COVID‐19, which can be life‐threatening for immunocompromised patients. Thus, further investigation is warranted.

## Graphical abstract

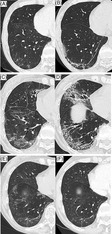

## INTRODUCTION

Coronavirus disease 2019 (COVID‐19), caused by severe acute respiratory syndrome coronavirus 2 (SARS‐CoV‐2), quickly spread globally, becoming a pandemic in the year of its emergence. Prolonged COVID‐19 has been reported in immunocompromised patients,^1^ especially in lymphoma patients.^2^ In particular, patients receiving chemotherapy containing B‐cell‐depleting agents, such as rituximab and bendamustine, have a higher incidence of prolonged COVID‐19.^3^, ^4^ Secondary interstitial pneumonitis treated after the resolution of COVID‐19 has also been reported.^5^, ^6^ Radiologically, more than half of persistent, post‐COVID‐19 interstitial lung disease cases have the pattern of organizing pneumonia chiefly presenting a dense consolidation in the subpleural and peribronchial areas. Patients with this condition have responded well to corticosteroid therapy.^7^

Herein, we report a case of COVID‐19 persisting longer than 11 months which developed interstitial changes in the lungs that were recalcitrant to corticosteroid therapy.

## CASE REPORT

A 69‐year‐old, female patient presented with a sore throat and high fever. Her elder brother, who was living with her, received a diagnosis of COVID‐19, and a PCR test of the patient's nasopharyngeal specimen for SARS‐AoC‐2 returned positive. She had a history of follicular lymphoma and had received chemotherapy consisting of rituximab and bendamustine, which is known to suppress cellular immunity highly, until 4 months before her current presentation. Thereafter she achieved remission of her lymphoma.

The patient had received three COVID‐19 vaccinations, with the last of which was 2 months before the current presentation. One month before COVID‐19 onset, her lymphocyte count was already low at 500/μL, and her serum IgG was 763 mg/dL, suggesting that her chemotherapy had caused immunosuppression. Hospital admission was considered because of her immunocompromised state, but as she had no respiratory failure or dyspnea, she chose not to be admitted at the time of her COVID‐19 diagnosis. Two months later, she was referred to our hospital again for a prolonged, high fever and was admitted. On admission, she had appetite loss, dysosmia, WBC count of 1500/μL, and neutrophils 1240/μL. Her lymphocytes had decreased to 150/μL, and her serum IgG was 681 mg/dL. A chest x‐ray revealed consolidation in the bilateral lower lungs.

The patient received remdesivir (RDV) and methyl‐prednisolone (m‐PSL) 20 mg.

**FIGURE 1:**
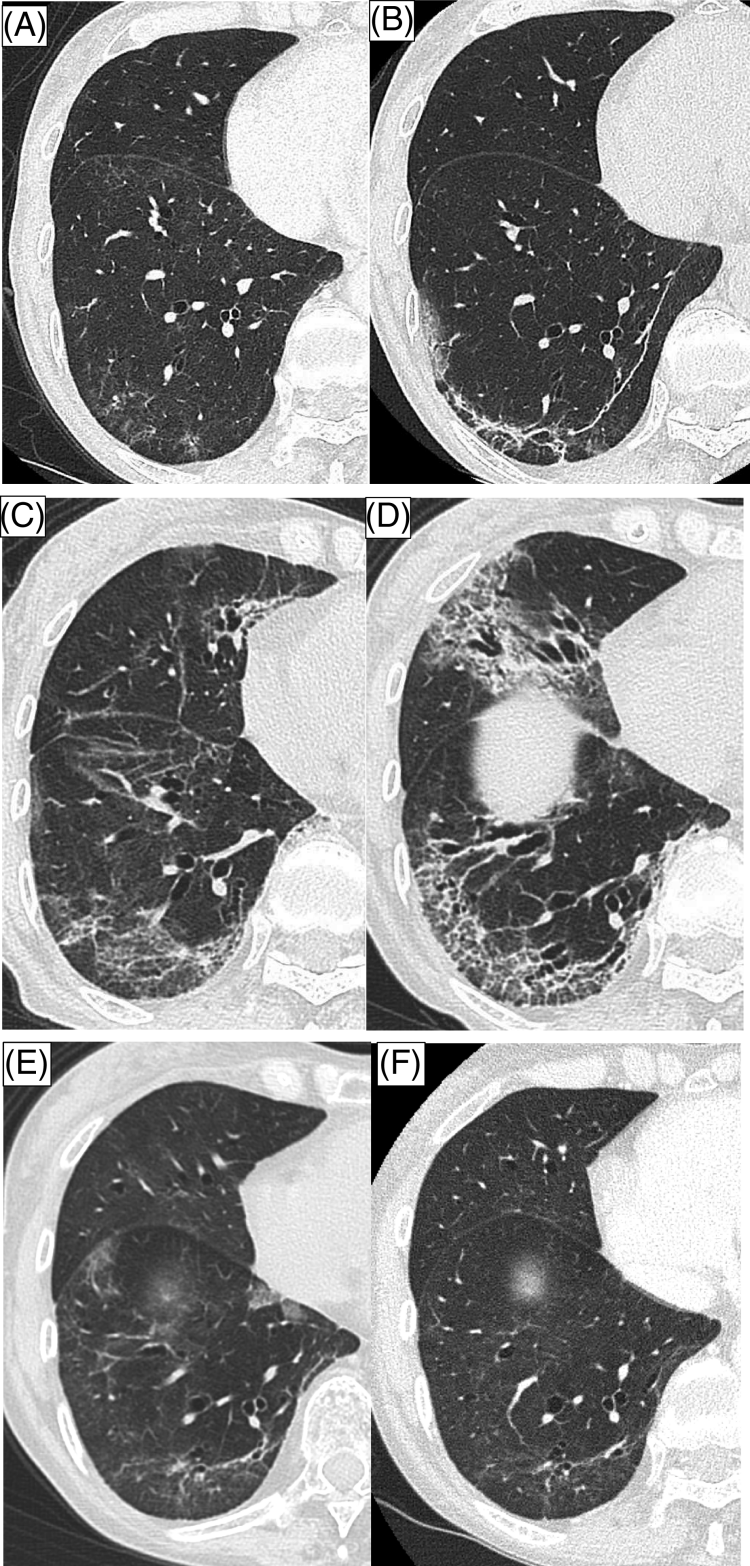
Thin‐slice computed tomography of the left lower lobe in chronological order. (A) Progressive infiltration of bilateral, patchy ground‐glass opacities (GGO) at the onset of COVID‐19. (B) New appearance of GGO after 2 months from the onset. (C) Bronchial traction appeared after the m‐PSL dosage was reduced <30 mg. (D) Progression of the GGO and reticular shadow after 8 months from the onset. (E) Improvement of the GGO after initiation of tacrolimus. (F) Resolution of reticular shadow after 14 months from the onset.

Her symptoms and bilateral consolidation on chest x‐ray improved at once, but a fever developed again. x‐Ray and computed tomography (CT) on hospital day 12 revealed progressive, bilateral, patchy ground‐glass opacities (GGO) (Figure 1A). No interstitial lung changes were evident on CT before COVID‐19 onset.

The m‐PSL dosage was increased to 80 mg, then gradually tapered. The patient was discharged after receiving oral prednisolone (PSL) 40 mg and atovaquone 1500 mg as prophylaxis against *Pneumocystis jirovecii* pneumonia.

**FIGURE 2:**
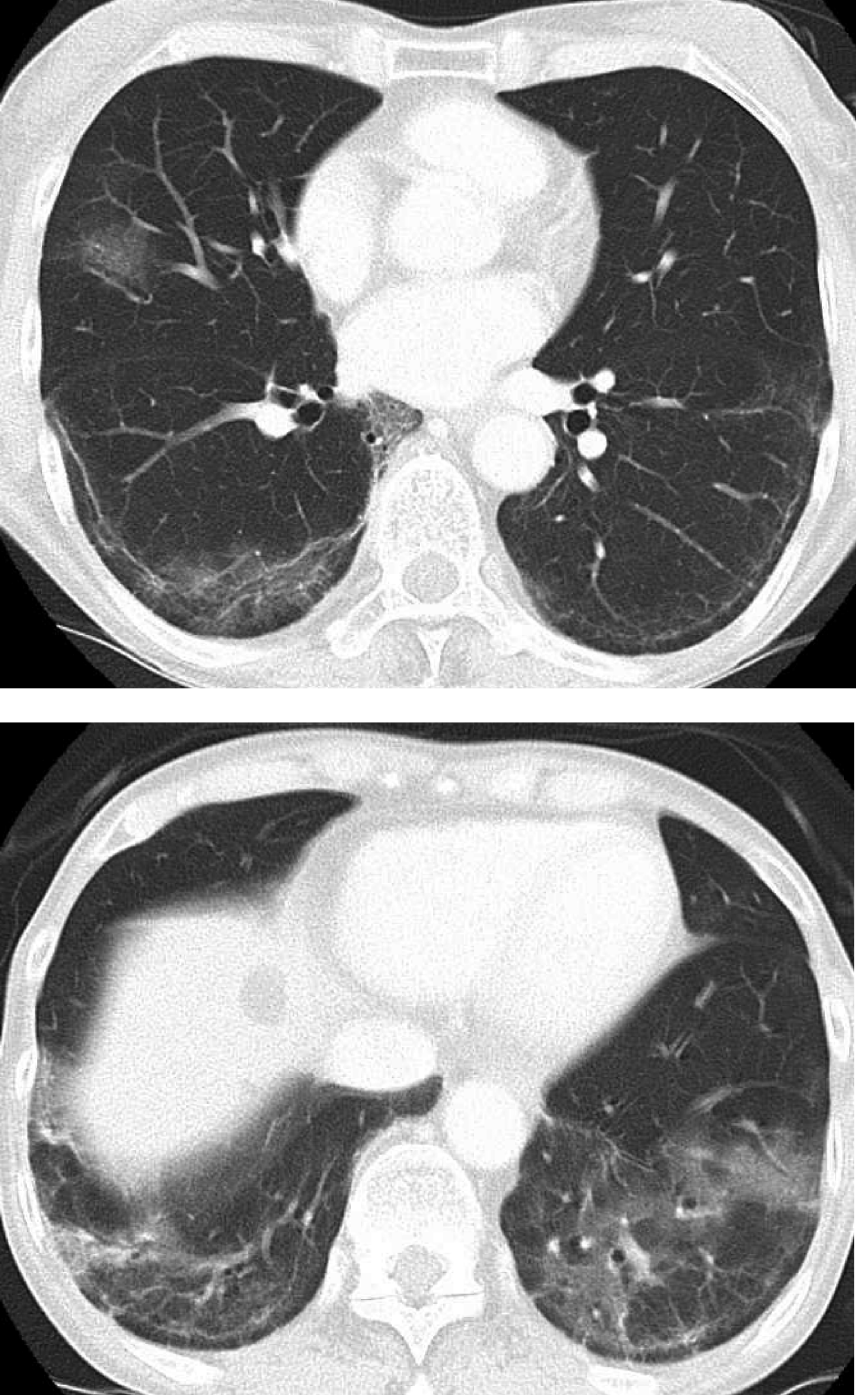
Computed tomography after 2 months from the onset.

Two months later, when the PSL dosage was 17.5 mg, a high fever developed again. The patient was positive for SARS‐CoV2 on PCR with a cycle threshold (Ct) value of 10.8 and 18.5 for the N2 and E gene, respectively. A 10‐day course of RDV and m‐PSL 35 mg was begun for a SARS‐CoV2 reinfection. CT revealed the appearance of new GGO in the bilateral lower lobes (Figures 1B and 2).

Four months after COVID‐19 onset, when the m‐PSL dosage had been reduced to less than 30 mg, the GGO of the bilateral lower lobes expanded to the upper lobes, and bronchial traction appeared (Figure 1C). Anti‐nuclear antibody, anti‐aminoacyl tRNA synthetase, including melanoma differentiation‐associated gene 5 (MDA‐5), rheumatoid factor, and antineutrophil cytoplasmic antibody were negative. PCR for SARS‐CoV‐2 still returned positive, and the Ct value was 20.4 and 19.4 for the N2 and E gene, respectively. Therefore, another anti‐viral agent, mirtazapine, was given with the aim of reducing the viral load.

**FIGURE 3:**
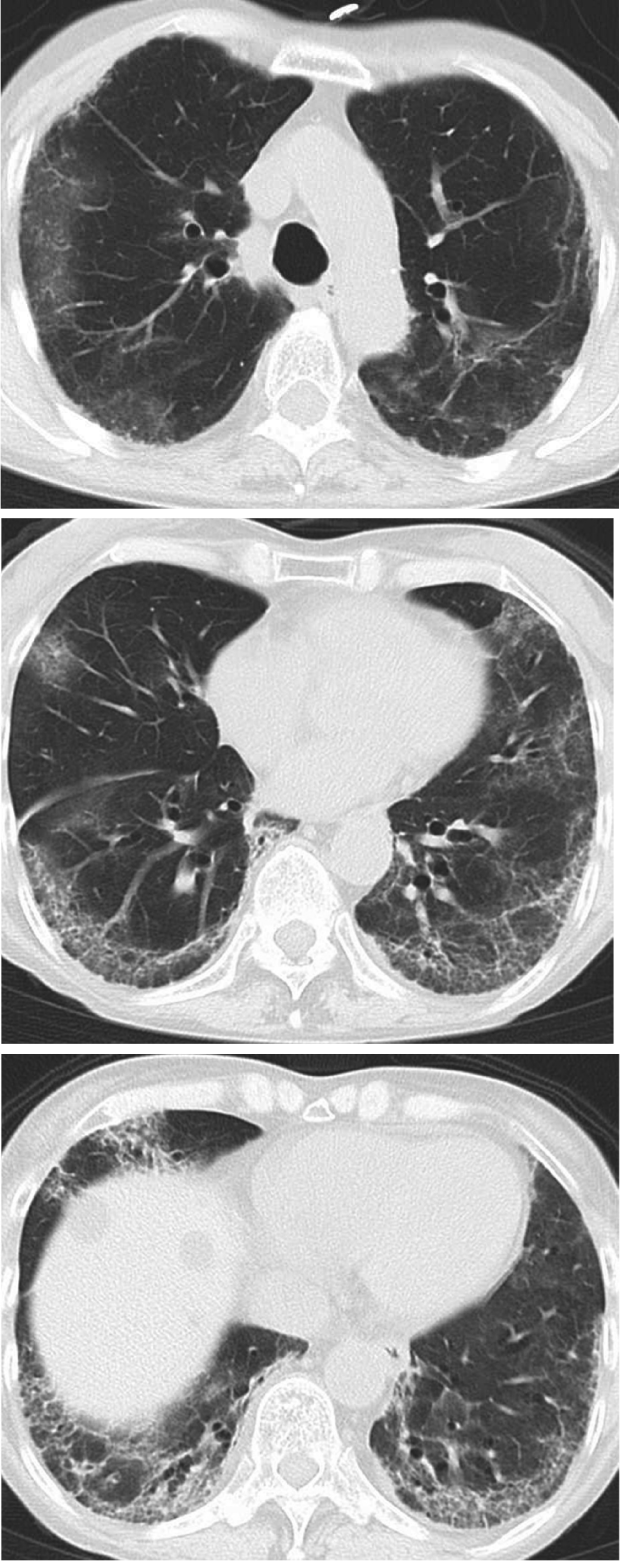
Computed tomography after 8 months from the onset.

Eight months after COVID‐19 onset, the patient was re‐admitted for fever and worsening dyspnea. PCR for SARS‐CoV‐2 was still positive, and the Ct values were similar to the previously obtained values. CT revealed GGO progression and a reticular shadow (Figures 1D and 3). After admission, she received remdesivir, m‐PSL 40 mg, and unfractionated heparin. She presented type 1 respiratory failure requiring 1 L/min of oxygen via a nasal cannula. On day 2, tocilizumab, an IL‐6 inhibitor, was administered for COVID‐19‐related pneumonia with respiratory failure. In addition, A further multidisciplinary discussion with pulmonologists and radiologist for a major referral hospital with subspecialty expertise took place to guide the choice of a steroid‐sparing agent. We discussed the radiological and clinical futures of prolonged pneumonitis after infection of COVID‐19 in patients with lymphoma and have undertaken Rituximab therapy resemble those of patients with rapidly progressive interstitial lung disease associated with MDA‐5. Thus, based on supporting evidence, the decision was made to administer tacrolimus, which has been approved in Japan as a treatment for rapidly progressive interstitial lung disease associated with MDA‐5. Oral tacrolimus 2 mg twice daily for a total dosage of 0.078 mg/kg/day was therefore begun on Day 3.

The patient became afebrile after the start of treatment, and her C‐reactive protein (CRP) value dropped from 6.10 ng/dL to 0.80 mg/dL on Day 5. Oxygen therapy was stopped on Day 12, and the bilateral shadows on the chest x‐ray also showed improvement. The PSL dosage was decreased to 20 mg on Day 22, and the patient was discharged on Day 23. Chest CT on Day 30 demonstrated widespread improvement of the GGO (Figure 1E).

**FIGURE 4:**
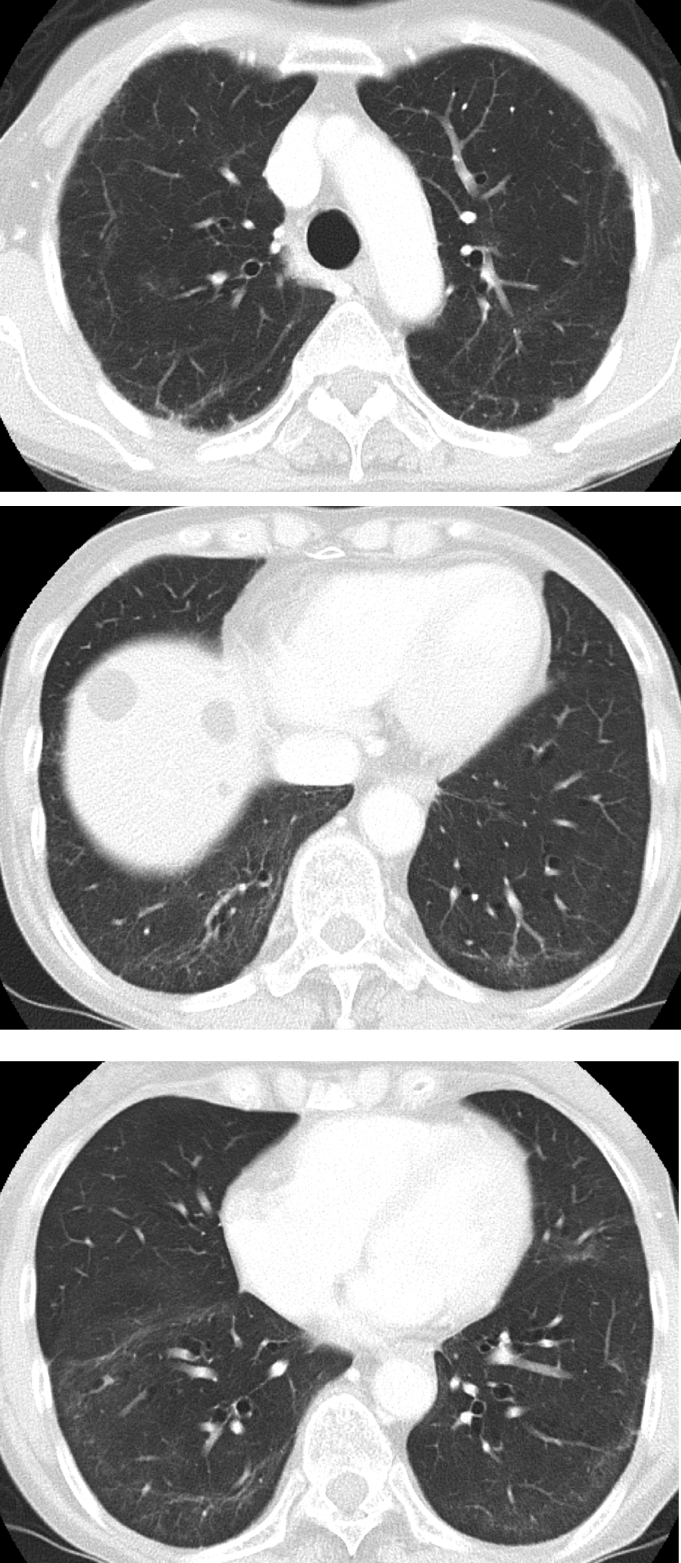
Computed tomography after 14 months from the onset.

She is currently receiving PSL 6 mg, which will gradually be tapered, and tacrolimus 4 mg 14 months after COVID‐19 onset. As a result, the reticular shadow has decreased (Figures 1F and 4).

## DISCUSSION

Historically, pulmonary fibrosis is known to develop after viral pneumonia.^8^ The mechanism of pulmonary fibrosis following COVID‐19 is thought to be as follows: SARS‐CoV‐2 damages the alveolar epithelium, inducing the production of inflammatory and immune cytokines and causing lung injury. The basement membrane becomes denudated, and interstitial fibroblasts migrate and proliferate in the alveolar space, leading to fibrosis.^9^

In the present case, positive PCR findings with Ct values around 20 persisted because the patient's cell‐mediated immunity had been suppressed by previous chemotherapy with rituximab and bendamustine. A persistent, viral infection of the pulmonary epithelial cells might induce a persistent release of inflammatory cytokines and cause lung injury relapse. Reducing the viral load significantly in this case using anti‐viral agents was challenging, but her Ct values were relatively high, indicating that the viral load was not high or that the viruses were inactive. Based on these considerations, the decision was made to administer immunosuppressive agents to reduce the inflammatory cytokines. As a result, the systemic inflammation and interstitial changes in the lungs improved despite the PCR results remaining positive and the Ct values showing little or no change.

Infection is a common cause of organizing pneumonia (OP), and viruses, such as the influenza virus and human immunodeficiency virus, can cause OP.^10^ OP and post‐COVID‐19 pulmonary sequelae (PCPS) consist of interstitial lung damage following COVID‐19 which responds well to corticosteroid therapy.^11^ Moreover, Katerine et al. reported that persistent interstitial lung disease following COVID‐19 occurred in 4.8% (35/837) of survivors, 30 of whom received corticosteroid treatment, which induced rapid and significant improvement and was well tolerated.^7^

Nonetheless, some cases are refractory to corticosteroid therapy, and there are a number of reports discussing the addition of immunosuppressive agents, such as mycophenolate moteril.^11^ There are some reports of prolonged pneumonitis, particularly among patients with lymphoma, which were refractory to treatment with anti‐SARS‐CoV‐2 antibodies or corticosteroids.^12^

Tacrolimus, a calcineurin inhibitor, is used as an immunosuppressive agent in Japan for patients who have received organ transplantation or have interstitial pneumonia associated with polymyositis or dermatomyositis. We thought that tacrolimus was able to suppress T‐cell mediated pro‐inflammatory cytokines and play a role of steroid‐sparing agent because of the similarity of the patient's disease profile with that of amyopathic dermatomyositis (CADM) associated with anti‐melanoma differentiation‐associated gene 5 (MDA‐5).^13^ Tacrolimus has specific mechanism to impair lymphocyte function and decrease pro‐inflammatory cytokines, and has an important role in the treatment of these diseases.^13^ Accordingly, in cell line, it has been shown that tacrolimus inhibits the immunophilin pathway which SARS‐Co‐V replication depends on.^14^ One case of interstitial pneumonitis after COVID‐19 treated with tacrolimus has been reported,^15^ and a randomized, phase 2 clinical trial evaluating the efficacy and safety of a combination therapy of methylprednisolone and tacrolimus for severe pneumonia secondary to COVID‐19 is currently underway.^13^ Our patient responded partially to the corticosteroid therapy but experienced a relapse when the PSL dosage was reduced to <30 mg. However, after the initiation of tacrolimus and tocilizumab therapy, the PSL dosage was able to be reduced to 6 mg.

It is unknown whether this treatment strategy is effective for other cases of persistent COVID‐19 accompanied by pneumonitis. In the early stages of the disease, immunosuppression might increase the viral load and worsen the symptoms, but the standard Ct value indicating the point at which an immunosuppressive agent ceases to be harmful is unclear. In the present case, attention should be given to secondary infections caused by immunosuppression as well as reactivation of COVID‐19. Even though the mortality rate associated with COVID‐19 has decreased, for immunocompromised patients, COVID‐19 can be still life‐threatening. Further study is needed to establish a treatment strategy for persistent pneumonitis in patients with a prolonged viral infection with impaired cellular immunity.

## AUTHOR CONTRIBUTIONS

Makiko Yomota wrote the first draft and Masaru Tanaka, Takayuki Kobayashi, Masatake Kitano, Saori Ikeda, Yusuke Kanemasa, Noriyo Yanagawa, Yukio Hosomi revised the article for important intellectual content. All authors approved the final version.

## CONFLICT OF INTEREST STATEMENT

Dr. Hosomi reports personal fees from AstraZeneca outside the submitted work; Dr. Yomota, Dr Tanaka, Dr Kobayashi, Dr Kitano, Dr Ikeda, Dr Kanemasa and Dr Yanagawa have nothing to disclose.

## ETHICS STATEMENT

We obtained written informed consent from the patient.

## Data Availability

The data that support the findings of this study are available on request from the corresponding author. The data are not publicly available due to privacy or ethical restrictions.
